# An Approximation Solution to Refinery Crude Oil Scheduling Problem with Demand Uncertainty Using Joint Constrained Programming

**DOI:** 10.1155/2014/730314

**Published:** 2014-03-16

**Authors:** Qianqian Duan, Genke Yang, Guanglin Xu, Changchun Pan

**Affiliations:** ^1^Department of Automation, Shanghai Jiao Tong University, Dongchuan Road 800, Shanghai, China; ^2^College of Mathematics and Information, Shanghai Lixin University of Commerce, China

## Abstract

This paper is devoted to develop an approximation method for scheduling refinery crude oil operations by taking into consideration the demand uncertainty. In the stochastic model the demand uncertainty is modeled as random variables which follow a joint multivariate distribution with a specific correlation structure. Compared to deterministic models in existing works, the stochastic model can be more practical for optimizing crude oil operations. Using joint chance constraints, the demand uncertainty is treated by specifying proximity level on the satisfaction of product demands. However, the joint chance constraints usually hold strong nonlinearity and consequently, it is still hard to handle it directly. In this paper, an approximation method combines a relax-and-tight technique to approximately transform the joint chance constraints to a serial of parameterized linear constraints so that the complicated problem can be attacked iteratively. The basic idea behind this approach is to approximate, as much as possible, nonlinear constraints by a lot of easily handled linear constraints which will lead to a well balance between the problem complexity and tractability. Case studies are conducted to demonstrate the proposed methods. Results show that the operation cost can be reduced effectively compared with the case without considering the demand correlation.

## 1. Introduction

In recent years refineries have to explore all potential cost-saving strategies due to intense competition arising from fluctuating product demands and ever-changing crude prices. Scheduling of crude oil operations is a critical task in the overall refinery operations [1–3].

Basically, the optimization of crude oil scheduling operations consists of three parts [4]. The first part involves the crude oil unloading, mixing, transferring, and multilevel crude oil inventory control process. The second part deals with fractionation, reaction scheduling, and a variety of intermediate product tanks control. The third part involves the finished product blending and distributing process. In this paper, we focus on the first part, as it is a critical component for refinery scheduling operations.

The crude oil scheduling problem has received considerable attention from researchers and different models have been developed on the basis of deterministic mathematical programming techniques. Lee et al. [5] developed a mixed-integer linear programming (MILP) model to solve a short-term crude oil scheduling problem, in which the linearity of the bilinear constraints is maintained by replacing bilinear terms with individual component flows, but it can lead to composition discrepancy. To overcome the composition discrepancy problem, Wenkai et al. [6] and Reddy et al. [7] proposed an iterative MIP-NLP model and an iterative discrete-time MIP model, respectively. However the models rely on time discretization representations. Recently, most mathematical models of crude oil scheduling operations put emphasis on continuous-time formulation so as to shorten the gap between theoretical research and real-world operation. Chryssolouris et al. [8] studied the similar problem as Lee et al. [5] and took the temperature cut-points into consideration for each distillation. Jia and Kelly [4] formulated the same problem by a state-task-network (STN) continuous-time representation. Hu and Zhu [9] extended the event-based model of Jia et al., [10, 11] to the slot model, by eliminating the redundant event points on others, reducing the size of the model, and hence the solution time of the problem.

Most of the current plant planning and scheduling models are based on deterministic programming. However, due to the volatile raw material prices, fluctuating products demands, and other changing market conditions, many parameters in a planning and scheduling model are usually uncertain. Neiro and Pinto [12] constructed a corporate planning model for multiple refineries using scenario based approach. Neiro and Pinto [12] developed a multiperiod MINLP model to deal with uncertainty in product price and crude price. However the scenario based approach provides no obvious information on the relation between reliability and profitability, which is crucial for decision makers. Several recent papers applied chance constrained programming models to the refinery short-term crude oil scheduling problem [13–16].

All of the aforementioned models in the domestic refinery optimization are either deterministic [5] or stochastic with independent demand distribution [14, 15]. Cao et al. [15] considered the demand correlation for different crude mix in the same time period. However, they did not consider the correlation for the same crude mix in different time periods. In this paper we will consider the crude mix demand correlation in demands not only for different crude mix in the same time period but also for the same crude mix indifferent time periods. These considerations have practical significance. For example, if two crude mixes are predominantly used as raw materials in another process, their demands will be positively correlated in each time period. Alternatively, unusually high demand for a crude mix in one time period more often than not is followed by lower than normal demand in the next period, implying negative correlation. Taking into account such information, whenever it is available, enables a more efficient allocation of crude mix capacity to minimize cost and meet certain marketing objectives.

In this paper, we will propose a stochastic multiperiod model with considering the uncertain crude mix demand correlations. The model employs two-level time structure formulation in which the entire scheduling horizon is divided into several interrelated macroperiods. Each macroperiod with fluctuation demand consists of time intervals with fixed length. A chance constrained programming formulation is developed for solving the problem. The deterministic form of the stochastic constraint is used in solving the problem iteratively. In real-world situations, the future demand always changes as time rolls forward. To deal with the uncertainty of the model, it is important to adjust the planning policy and update the corresponding schedule and the correlation structure of the demand at the end of each time period based on the real sales [17].

The rest of the paper is organized as follows. The scheduling problem is specified in Section 2. In Section 3, a stochastic multiperiod model with two-level time structure formulation is formulated. In Section 4, the deterministic representation of the model is presented. In Section 5, an approximation method combining relax-and-tight technique is developed to solve the joint chance constrained problem above. By relax, we mean that the ΣA approach [18] is used to approximate the original-covariance matrix with a new one of simplified covariance structure. By tight, we mean that the joint chance constrains are transformed into several linear constraints with parameterized dependent which are more stringent than the original constraints. Moreover, an update policy upon the realization of the random demands of crude mix is described. A test problem involving correlated random crude mix demands is solved in Section 6, highlighting various modeling and algorithmic issues.  Section 7 summarizes the work and provides some concluding remarks.

## 2. Problem Statements and Operation Rules

### 2.1. Problem Statements

**Figure 1:**
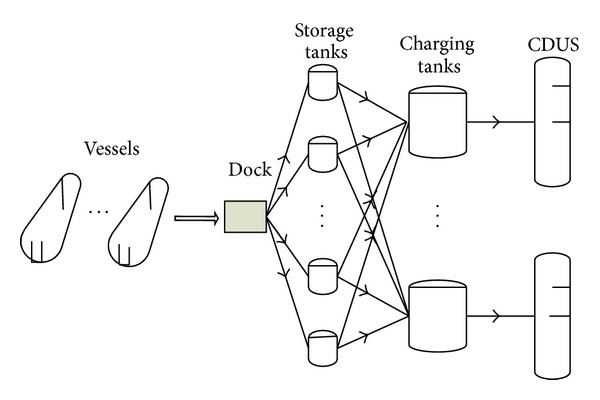
Typical flow process for the refinery crude oil operations.

The problem studied involves crude oil unloading process from vessels to storage tanks, transferring process from storage tanks to charging tanks (where several crude oils are mixed) and charging process from charging tanks to crude oil distillations (CDUs).  Figure 1 shows the typical processes. During a given scheduling horizon, crude vessels arrive in the vicinity of the refinery docking station and, according to FCFS, wait for unloading of the preceding vessel in the docking station. At the docking station, crude oil is unloaded into storage tanks. Crude oil is then transferred from storage tanks to charging tanks which are buffers to produce a crude mix, of which component compositions were determined at the planning level. The crude oil mix in each charging tank is then charged into a CDU. Given the configuration of the multistage system as well as the uncertain arrival times of vessels, equipment capacity limitations, and key component concentration ranges, the problem is then to determine the following operating variables to minimize operating costs: (a) waiting time of each vessel at sea, (b) unloading time of each vessel, (c) crude unloading rate from vessels to storage tanks, (d) crude oil transfer and mixing rate from storage tank to charging tanks, (e) inventory levels of storage and charging tanks, (f) CDU charging rates, and (g) sequence for charging mixed crude into each CDU.

A basic deterministic model assumption was presented in [5]. However, in this paper we will extend it to a new mixed-integer nonlinear stochastic programming model with chance constraints. The demands for different crude mix are uncertain and possibly correlated, reflecting changing market conditions and periodic variation in customer orders. An optimal planning policy for minimizing expected cost is developed. This is achieved by letting crude mix demands be satisfied with at least a prespecified probability level.

The proposed stochastic model involves the following features and assumptions.

1. A two-level time structure introduced by Fleischmann and Meyr [19] is adopted for modeling a general stochastic system. The entire planning interval is divided into macroperiods each with fluctuation demand and each macroperiod consists of time intervals with fixed length.
2. The demands for different crude mixes are modeled as multivariate normally distributed random variables. The normality assumption has been widely invoked in the literature because it captures the essential features of demand uncertainty and it is convenient to use. The use of more “complex” probability distributions is hindered by the fact that statistical information apart from mean and covariance estimates of product demands is rarely available.
3. The demand correlation, in the problem formulation, stands for correlation of demands for different crude mixes in the same time period and demands for the same crude mix in different time periods.
4. The penalty for some crude mix shortfalls is proportional to the amount of underproduction.

### 2.2. Operation Rules

The operation rules are shown as follows: (1) the refinery uses only one docking station, and a new arriving vessel has to wait at sea until the anterior vessel leaves the docking station; (2) while a charging tank is charging CDU, crude oil from the storage tanks cannot be fed into the charging tank and vice versa; (3) each charging tank can only charge one CDU at each time interval; (4) each CDU can only be charged by one charging tank at each time interval; (5) CDUs must be operated continuously throughout the scheduling time horizon.

## 3. Mathematical Model

*Indices and Sets*

 
*i* ∈ {1,…, *N* ^*S*^}  = crude oil storage tank and the crude oil in it.
 
*j* ∈ {1,…, *N* ^*B*^}  = crude oil charging tank and the crude oil mix in the charging tank.
 
*k* ∈ {1,…, *N* ^*C*^}  = key component of crude oil.
 
*l* ∈ {1,…, *N* ^CDU^}  = crude distillation unit.
 
*m* ∈ {1,…, *N* ^*M*^} = macroperiod.
 
*t* ∈ {1,…, *N* ^SCH^}  = time interval, *N* ^SCH^, denotes scheduling horizon.
 
*T*_*m*_  = the starting time of the macroperiod* m. *
 
*v* ∈ {1,…, *N* ^*V*^}  = crude vessels.

*Variables*

 
*D*_*j*,*l*,*t*_  = 0-1 variable to denote if the crude oil mix in charging tank *j* charges CDU *l* at time* t. *
 
*X*_*v*,*t*_^*F*^  = 0-1 variable to denote if vessel *v* starts unloading at time* t. *
 
*X*_*v*,*t*_^*L*^  = 0-1 variable to denote if vessel *v* just completes unloading at time* t. *
 
*X*_*v*,*t*_^*W*^ = 0-1 continuous variable to denote if vessel* v* is unloading its crude oil at time* t. *
 
*Z*_*j*,*j*′,*l*,*t*_ = 0-1 continuous variable to denote if transition from crude mix (or charging tank)* j* to  *j*′  at time* t* in CDU* l. *
 
*f*_*i*,*j*,*k*,*t*_^SB^  = volumetric flow rate of component *k* from storage tank *i* to charging tank *j* at time* t. *
 
*f*_*j*,*l*,*k*,*t*_^BC^  = volumetric flow rate of component *k* from charging tank *j* to CDU *l* at time* t. *
 
*F*_*v*,*i*,*t*_^VS^  = volumetric flow rate of crude oil from vessel *v* to storage tank *i* at time* t. *
 
*F*_*i*,*j*,*t*_^SB^  = volumetric flow rate of crude oil from storage tank *i* to charging tank *j* at time* t. *
 
*F*_*j*,*l*,*t*_^BC^  = volumetric flow rate of crude oil mix from charging tank *j* to CDU *l* at time* t. *
 
*T*_*v*_^*F*^ = vessel* v* unloading initiation time.
 
*T*_*v*_^*L*^  = vessel* v* unloading completion and departure time.
 
*v*_*j*,*k*,*t*_^*B*^  = volume of component* k* in charging tank* j* at time* t. *
 
*V*_*v*,*t*_^*V*^  = volume of crude oil in crude vessel* v* at time* t. *
 
*V*_*i*,*t*_^*S*^  = volume of crude oil in storage tank* i* at time* t. *
 
*V*_*j*,*t*_^*B*^  = volume of mixed oil in charging tank* j* at time* t. *

*Parameters*

 
*C*_*V*_^UNLOAD^  = unloading cost of vessel *v* per time interval.
 
*C*_*V*_^SEA^  = sea waiting cost of vessel *v* per time interval.
 
*C*_*i*_^INVST^  = inventory cost of storage tank *i* per time per volume.
 
*C*_*j*_^INVBL^ = inventory cost of charging tank *j* per time per volume.
 
*C*_*j*,*j*′_^SETUP^ = changeover cost for transition from crude mix* j* to  *j*′ in CDU.
 
*C*_*j*_^PEN^ = penalty cost for crude mix *j* shortfalls.
 
*D*_*j*,*m*_^*M*^ = stochastic demand of crude mix *j* by CDUs during the macroperiod* m. *
 
*F*_*v*,*i*_^VS,min⁡^  = minimum crude oil transfer rate from vessel *v* to storage tank* i. *
 
*F*_*v*,*i*_^VS,max⁡^  = maximum crude oil transfer rate from vessel *v* to storage tank* i. *
 
*F*_*i*,*j*_^SB,min⁡^ = minimum crude oil transfer rate from storage tank *i* to charging tank* j. *
 
*F*_*i*,*j*_^SB,max⁡^  = maximum crude oil transfer rate from storage tank *i* to charging tank* j. *
 
*F*_*j*,*l*_^BC,min⁡^  = minimum crude oil charging rate from charging tank *j* to CDU* l. *
 
*F*_*j*,*l*_^BC,max⁡^ = maximum crude oil charging rate from charging tank *j* to CDU* l. *
 
*T*_*v*_^ARR^ = crude vessel *v* arrival time around the docking station.
 
*V*_*v*,0_^*V*^  = initial volume of crude oil in crude vessel* v. *
 
*V*_*i*_^*S*,min⁡^ = minimum crude oil volume of storage tank* i. *
 
*V*_*i*_^*S*,max⁡^  = maximum crude oil volume of storage tank* i. *
 
*V*_*i*,0_^*S*^  = initial crude oil volume of storage tank* i. *
 
*V*_*j*_^*B*,min⁡^ = minimum mixed crude oil volume of charging tank* j. *
 
*V*_*j*_^*B*,max⁡^ = maximum mixed crude oil volume of charging tank* j. *
 
*ε*_*i*,*k*_^*S*^ = concentration of component *k* in the crude oil of storage tank* i. *
 
*ε*_*j*,*k*,0_^*B*^  = initial concentration of component *k* in the crude mix of charging tank* j. *
 
*ε*_*j*_^*B*,min⁡^  = minimum concentration of component *k* in the crude mix of charging tank* j. *
 
*ε*_*j*_^*B*,max⁡^ = maximum concentration of component *k* in the crude mix of charging tank* j. *

In this paper, a two-level time structure is used to model a general dynamic production system. We consider the crude mix *j* to be scheduled over a finite planning horizon consisting of macroperiods *m* = 1 ⋯ *N* ^*M*^. Each macroperiod *m* is divided into time intervals with fixed length, where  *T*_*m*_  represents the starting time of macroperiod *m*. Let  *D*_*j*,*m*_^*M*^, a random variable, denote the demand of crude mix *j* in macroperiod *m*. We approximate the crude mix demand as a multivariate normal distribution with a specific correlation structure  *D* ^*M*^ ~ *N*(*μ*, Σ)  with covariance matrix  Σ, where  *D* ^*M*^≝[*D*_*j*,*m*_^*M*^], *μ*≝[*μ*_*j*,*m*_]. It means that the demands for different mix crudes not only are correlated but also are the demands for the same mixed crude in different macroperiods.

The mathematical model is formulated as follows.

(*1) Operating Cost *

 Minimize:
 Subject to:

*(2) Vessel Arrival and Departure Operation Rules. *Each vessel arrives at the docking station for unloading only once throughout the scheduling horizon:

(2a) $$\begin{array}{c} \sum_{t=1}^{N^{\text{SCH}}}X_{v,t}^{F}=1\;\forall v. \end{array}$$

Each vessel leaves the docking station only once throughout the scheduling horizon. Consider

(2b) $$\begin{array}{c} \sum_{t=1}^{N^{\text{SCH}}}X_{v,t}^{L}=1\;\forall v. \end{array}$$

Equation for unloading initiation time:

(2c) $$\begin{array}{c} T_{v}^{F}=\sum_{t=1}^{N^{\text{SCH}}}tX_{v,t}^{F}\;\forall v. \end{array}$$

Equation for unloading completion time:

(2d) $$\begin{array}{c} T_{v}^{L}=\sum_{t=1}^{N^{\text{SCH}}}tX_{v,t}^{L}\;\forall v. \end{array}$$

Each crude vessel should start unloading after arrival time set in the planning level:

(2e) $$\begin{array}{c} T_{v}^{F}\geq T_{v}^{\text{ARR}}\;\forall v. \end{array}$$

Duration of the vessel unloading is bounded by the initial volume of oil in the vessel divided by the maximum unloading rate:

(2f) $$\begin{array}{c} T_{v}^{L}-T_{v}^{F}\geq \left\lceil \frac{V_{v,0}^{V}}{\max\left\lceil F_{v,i}^{VS,\max}\right\rceil }\right\rceil \;\forall v. \end{array}$$

⌈⌉  corresponds to round-up of the next highest integer value. Vessel in the sea cannot arrive at the docking station for unloading unless the preceding vessel leaves:

(2g) $$\begin{array}{c} T_{v+1}^{F}\geq T_{v}^{L}\;\forall v. \end{array}$$

Unloading is possible between time  *T*_*v*_^*F*^ and  *T*_*v*_^*L*^:

(2h) $$\begin{array}{c} X_{v,t}^{W}\leq \sum_{\tau =1}^{t}X_{v,\tau }^{F},\;\;X_{v,t}^{W}\leq \sum_{\tau =t}^{\text{SCH}}X_{v,\tau }^{L}\;\forall v,t. \end{array}$$

*(3) Material Balance Equations for the Vessel. *For vessel* v*, the oil volume at time *t* equals the initial volume minus transferred volume from vessel *v* to storage tanks up to time* t*:

(3a) $$\begin{array}{c} V_{v,t}^{V}=V_{v,0}^{V}-\sum_{i=1}^{N^{\text{ST}}}\sum_{\tau =1}^{t}F_{v,i,\tau }^{VS}\;\forall v,t. \end{array}$$

Operating constraints on crude oil transfer rate from vessel *v* to storage tank *i* at time* t*

(3b) $$\begin{array}{c} F_{v,i}^{\text{VS},\min}X_{v,t}^{W}\leq F_{v,i,t}^{\text{VS}}\leq \;F_{v,i}^{\text{VS},\max}X_{v,t}^{W}\;\;\forall v,i,t. \end{array}$$

The volume of crude oil transferred from vessel *v* to storage tanks during the scheduling horizon equals the initial crude oil volume of vessel* v*:

(3c) $$\begin{array}{c} \sum_{i=1}^{N^{\text{ST}}}\sum_{t=1}^{N^{\text{SCH}}}F_{v,i,t}^{\text{VS}}=V_{v,0}^{V}\;\forall v,i,t. \end{array}$$

*(4) Material Balance Equations for Storage Tanks. *The oil volume in storage tank *i* at time *t* equals the initial oil volume in tank *i* plus the oil volume transferred from vessels to tank *i* up to the time *t* and minus oil volume removed from tank *i* to charging tanks up to the time* t*:

(4a) Vi,tS=Vi,0S+∑v=1NV∑τ=1tFv,i,τVS−∑j=1NBT∑τ=1tFi,j,τSB ∀i,t

Operating constraints on crude oil transfer rate from storage tank *i* to charging tank *j* at time* t,* the term 1 − ∑_*l*=1_^NCDU^ *D*_*j*,*l*,*t*_ denotes that if charging tank *j* is charging any CDU, there is no oil transfer from storage tank *i* to charging tank* j*. Consider

(4b) Fi,jSB,min⁡(1−∑l=1NCDUDj,l,t) ≤Fi,j,tSB≤  Fi,jSB,max⁡(1−∑l=1NCDUDj,l,t) ∀i,j,t.

Volume capacity limitations for storage tank *i* at time* t*

(4c) $$\begin{array}{c} V_{i}^{S,\min}\leq V_{i,t}^{S}\leq V_{i}^{S,\max}\;\forall i,t. \end{array}$$

*(5) Material Balance Equations for Charging Tanks. *The crude oil mix in charging tank *j* at time *t* equals the initial oil volume in charging tank *j* plus the crude oil transferred from storage tanks to charging tank* j* up to time* t* and minus the crude oil mix *j* charged into CDUs up to time* t*:

(5a) Vj,tB=Vj,0B+∑i=1NST∑τ=1tFi,j,τSB −∑l=1NCDU∑τ=1tFj,l,τBC ∀j,t.

Operating constraints on mixed oil transfer rate from charging tank *j* to CDU *l* at time* t*

(5b) $$\begin{array}{c} {F_{j,l}^{\text{BC},\min}D}_{j,l,t}\leq F_{j,l,t}^{\text{BC}}\leq F_{j,l}^{\text{BC},\max}D_{j,l,t}\;\forall j,l,t. \end{array}$$

Volume capacity limitations for charging tank *j* at time* t*

(5c) $$\begin{array}{c} V_{j}^{B,\min}\leq V_{j,t}^{B}\leq V_{j}^{B,\max}\;\forall j,t. \end{array}$$

*(6) Material Balance Equations for Component k* *in Charging Tanks. *The volume of component *k* in charging tank *j* at time *t* equals the initial component *k* in charging tank *j* plus component *k* in crude oil transferred from storage tanks to charging tank *j* up to the time *t* and minus component *k* in crude oil mix *j* transferred to CDUs up to the time* t*:

(6a) $$\begin{array}{c} v_{j,t}^{B}=v_{j,0}^{B}+\sum_{\tau =1}^{t}\sum_{i=1}^{N^{\text{ST}}}f_{i,j,\tau }^{\text{SB}}-\sum_{\tau =1}^{t}\sum_{l=1}^{N^{\text{CDU}}}f_{j,l,\tau }^{\text{BC}}\;\forall j,k,t. \end{array}$$

Operating constraints on volumetric flow rate of component *k* from storage tank *i* to charging tank* j *

(6b) $$\begin{array}{c} f_{i,j,k,t}^{\text{SB}}=F_{i,j,t}^{\text{SB}}\varepsilon_{i,k}^{S}\;\forall i,j,k,t. \end{array}$$

Operating constraints on volumetric flow rate of component *k* from charging tank *j* to CDU* l*

(6c) $$\begin{array}{c} F_{j,l,t}^{\text{BC}}\varepsilon_{j,k}^{B,\min}\leq f_{j,l,k,t}^{\text{BC}}\leq F_{j,l,t}^{\text{BC}}\varepsilon_{j,k}^{B,\max}\;\forall j,l,k,t. \end{array}$$

Volume capacity limitations for component *k* in charging tank *j* at time* t*

(6d) $$\begin{array}{c} V_{j,t}^{B}\varepsilon_{j,k}^{B,\min}\leq v_{j,k,t}^{B}\leq V_{j,t}^{B}\varepsilon_{j,k}^{B,\max}\;\forall j,l,k,t. \end{array}$$

*(7) Operating Rules for Crude Oil Charging. *Charging tank *j* can charge at most one CDU at any time* t: *

(7a) $$\begin{array}{c} \sum_{l=1}^{N^{\text{CDU}}}D_{j,l,t}\leq 1\;\forall j,t. \end{array}$$

CDU *l* can be charged only by one charging tank at any time* t*:

(7b) $$\begin{array}{c} \sum_{j=1}^{N^{\text{BT}}}D_{j,l,t}=1\;\forall l,t. \end{array}$$

If CDU *l* is charged by crude oil mix *j* at time* t *− 1 and charged by  *j*′  at time* t*, changeover cost is involved:

(7c) Zj,j′,l,t≥Dj′,l,t+Dj,l,t−1−1j,j′(j≠j′)=1,…,NB ∀l,t.

The proposed formulation involves stochastic expressions, which requires a different course of action for transforming it into an equivalent deterministic form. The deterministic equivalent representation of the expectation of the objective function is examined in the next section.

## 4. Chance Constraint Based Deterministic Transformation 

### 4.1. Expectation of Penalty Cost

The expectation of the underproduction penalty cost term for crude mix *j* in macroperiod *m* is equal to

(8) $$\begin{array}{c} E\left[\sum_{j=1}^{N^{\text{BT}}}C_{j}^{\text{PEN}}\sum_{m=1}^{N^{M}}\max\left(0,D_{j,m}^{M}-\sum_{l=1}^{N^{\text{CDU}}}\sum_{T_{m}}^{T_{m+1}-1}F_{j,l,t}^{\text{BC}}\right)\right], \end{array}$$

where

(9) Cj,m≜max⁡(0,Dj,mM−∑l=1NCDU∑TmTm+1−1Fj,l,tBC)=−min⁡⁡(0,∑l=1NCDU∑TmTm+1−1Fj,l,tBC−Dj,mM)=−[min⁡⁡(Dj,mM,∑l=1NCDU∑TmTm+1−1Fj,l,tBC)−Dj,mM].

To facilitate the calculation of the expectation, the standardization of the normally distributed variables *D*_*j*,*m*_^*M*^ and variables  ∑_*l*=1_^*N*^CDU^^∑_*T*_*m*__^*T*_*m*+1_−1^ *F*_*j*,*l*,*t*_^BC^  is performed first. Normal random variables can be recast into the standardized normal form, with a mean of zero and a variance of 1, by subtracting their mean and dividing by their standard deviation (square root of variance). This defines the standardized normal variables

(10) $$\begin{array}{c} x_{j,m}=\frac{D_{j,m}^{M}-\hat{D_{j,m}^{M}}}{\sigma_{j,m}}, \end{array}$$

where$\;\hat{D_{j,m}^{M}}\;$denotes the mean of *D*_*j*,*m*_^*M*^ and *σ*_*j*,*m*_ the square root of its variance. In the same way, the “standardization” of the variables  ∑_*l*=1_^*N*^CDU^^∑_*T*_*m*__^*T*_*m*+1_−1^ *F*_*j*,*l*,*t*_^BC^  defines

(11) $$\begin{array}{c} K_{j,m}=\frac{\sum_{l=1}^{N^{\text{CDU}}}\sum_{T_{m}}^{T_{m+1}-1}F_{j,l,t}^{\text{BC}}-\hat{D_{j,m}^{M}}}{\sigma_{j,m}}. \end{array}$$

Using the method in paper [17], the underproduction penalty term  *C*_*j*,*m*_  is charging into the following form:

(12) $$\begin{array}{c} C_{j,m}=-\left[-f\left(K_{j,m}\right)+\left(1-\Phi \left(K_{j,m}\right)\right)K_{j,m}\right], \end{array}$$

where  *f* is the standardized normal distribution function and Φ denotes the cumulative probability function of a standard normal random variable.

### 4.2. Satisfaction Level for Single Crude Mix Demand

The minimization of the objective function (1), as defined above, establishes the production and planned sales policy which most appropriately balances profits with inventory costs and underproduction shortfalls. A crude mix demand satisfaction level is not explicitly specified, but rather it is the outcome of the minimization of the profit function. While higher values of the parameter *C*_*j*_^PEN^ conceptually increase the probability of demand satisfaction, this strategy may still lead to unacceptably low probabilities of satisfying certain crude mix demands (see examples). Therefore, the setting of explicit probability targets on crude mix demand satisfaction is much more desirable. A systematic way to accomplish this is to impose explicit lower bounds on the probabilities of satisfying a single crude mix demand. This requirement for crude mix *j* in macroperiod *m* assumes the following form:

(13) $$\begin{array}{c} \text{Pr}\left[\sum_{l=1}^{N^{\text{CDU}}}\sum_{T_{m}}^{T_{m+1}-1}F_{j,l,t}^{\text{BC}}\geq D_{j,m}^{M}\right]\geq \beta_{j,m}. \end{array}$$

This constraint, known as a* chance constraint*, imposes a lower bound  *β*_*j*,*m*_  on the probability that the crude mix demand realization will be greater than the planned sales  *D*_*j*,*m*_^*M*^  for crude mix *j* in macroperiod *m*. The deterministic equivalent representation can be obtained based on the concepts introduced by Charnes and Cooper [20].

Specifically, by subtracting the mean and dividing by the standard deviation of  *D*_*j*,*m*_^*M*^, the chance constraint can be written equivalently as

(14) $$\begin{array}{c} \text{Pr}\left[\frac{\sum_{l=1}^{N^{\text{CDU}}}\sum_{T_{m}}^{T_{m+1}-1}F_{j,l,t}^{\text{BC}}-\hat{D_{j,m}^{M}}}{\sigma_{j,m}}\geq \frac{D_{j,m}^{M}-\hat{D_{j,m}^{M}}}{\sigma_{j,m}}\right]\geq \beta_{j,m},\\ \text{Pr}\left\lceil K_{j,m}\geq x_{j,m}\right\rceil \geq \beta_{j,m}. \end{array}$$

The right-hand side of the inequality within the probability sign is a normally distributed random variable with a mean of zero and a variance of 1. This implies that the chance constraint can be replaced with the following deterministic equivalent expression:

(15) $$\begin{array}{c} \phi \left(K_{j,m}\right)\geq \beta_{j,m}. \end{array}$$

The application of the inverse of the normal cumulative distribution function  *ϕ* ^−1^, which is a monotonically increasing function, yields

(16) $$\begin{array}{c} K_{j,m}\geq \phi^{-1}\left(\beta_{j,m}\right) \end{array}$$

Inspection of the deterministic equivalent constraint reveals that it is linear in the deterministic variables and is composed of the mean of the original constraint augmented by the squared root of its variance times *ϕ* ^−1^(*β*_*j*,*m*_).

### 4.3. Satisfaction Level for All Crude Mix Demand

In some cases a probability target is desired for the demand satisfaction of a group of crude mix in different macroperiod. For example, when only up to 10%, unsatisfied crude mix demand can be tolerated throughout the entire horizon without distinguishing between different crude mixes. While single-product chance constraints are unaffected by correlations between crude mix demands, this is not always the case for joint chance constraints.

This gives rise to joint chance constraints. Joint chance constraints impose a probability target of simultaneously satisfying the demands for a group of crude mixes in different macroperiods as follows:

(17) Pr[⋂(j,m)∈Im(∑l=1NCDU∑TmTm+1−1Fj,l,tBC≥Dj,mM)]≥βm   Im={(j,m′) ∣ 1≤j≤NBT,m≤m′≤M},

where  *I*_*m*_  is the set of product-period (*j*, *m*) combinations whose simultaneous demand satisfaction with probability of at least  *β*_*m*_  is sought and  *I*_*m*_ = {(*j*, *m*′) | 1 ≤ *j* ≤ *N* ^BT^, *m* ≤ *m*′ ≤ *M*} is the set of all joint chance constraint. The joint probability,  *β*_*m*_, is the probability of intersection of individual constraints to be satisfied.

We use the approach in [21] to solve the joint chance constrained problem. The joint chance constraint equation (17) can be written as separated equivalent deterministic equations. Initially we choose the *T* constraints in a manner such that together they are more stringent than (17). The initial set of individual linear constraints equation (18) replacing (17) was obtained using the following argument. First we denote the event ∑_*l*=1_^*N*^CDU^^∑_*T*_*m*__^*T*_*m*+1_−1^ *F*_*j*,*l*,*t*_^*BC*^ ≥ *D*_*j*,*m*_^*M*^  by  *A*_*j*,*m*_ and its complement event  ∑_*l*=1_^*N*^CDU^^∑_*T*_*m*__^*T*_*m*+1_−1^ *F*_*j*,*l*,*t*_^BC^ < *D*_*j*,*m*_^*M*^ by *A*_*j*,*m*_^*C*^. From Boole's inequality of probability theory [22] it is well known that

(18) $$\begin{array}{c} P\left[{⋃}_{(j,m)\in I_{m}}A_{j,m}\right]\leq \sum_{j=1}^{J}\sum_{m=1}^{M}P\left[A_{j,m}\right]. \end{array}$$

Thus it follows that if  *P*[*A*_*t*_^*C*^] ≤ (*β*_*m*_/*T*), *m* = 1,2,…, *M*, then

(19) P[⋂(j,m)∈ImAj,m]=1−P[⋃(j,m)∈ImAj,m]≥1−∑j=1J∑m=1MP[Aj,mC]≥1−(1−βm).

Now, because  *D*_*j*,*m*_^*M*^  is normally distributed with mean *μ*_*j*,*m*_ and standard deviation *σ*_*j*,*m*_, *P*[*A*_*j*,*m*_^*c*^] ≤ (1 − *β*_*m*_)/*T* is equivalent to *P*[*D*_*j*,*m*_^*M*^ > ∑_*l*=1_^*N*^CDU^^∑_*T*_*m*__^*T*_*m*+1_−1^ *F*_*j*,*l*,*t*_^BC^] ≤ (1 − *β*_*m*_)/*T*, which in its turn is equivalent to

(20) $$\begin{array}{c} \sum_{l=1}^{N^{\text{CDU}}}\sum_{T_{m}}^{T_{m+1}-1}F_{j,l,t}^{BC}\geq \mu_{j,m}+z_{\alpha /T}\sigma_{j,m}, \end{array}$$

where  *z*_*α*/*T*_  is the 100(1 − ((1 − *β*_*m*_)/*T*)th percentile of the standard normal distribution. Subsequently we will suppress the subscript for* z* and replace (20) by the following equation:

(21) $$\begin{array}{c} \sum_{l=1}^{N^{\text{CDU}}}\sum_{T_{m}}^{T_{m+1}-1}F_{j,l,t}^{BC}\geq \mu_{j,m}+z\sigma_{j,m}. \end{array}$$

The initial value of  *z*  will be chosen to be *z* = *z*_*α*/*T*_.

Using the individual constraints equation (21) results in a more conservative solution than the joint chance constraint equation (17). Therefore, it is required to update the value of  *𝓏*  in order to find a solution which satisfies the required confidence level. The method in updating  *𝓏*  proposed in [23] is used, which is based on the interpolation of  *𝓏*  values.

The deterministic forms of the objective function and stochastic constraint are used in solving the joint chance constrained problem iteratively by using a different  *𝓏*  value. The algorithm is examined in the next section.

## 5. The Algorithm and Update Policy for the Joint Chance Constrained Problem

In this section, a method is developed to solve the joint chance constrained problem above, and then an update policy upon the realization of the random demands of crude mix is described.

### 5.1. Algorithm for the Joint Chance Constrained Problem

The following three steps are performed iteratively.

**Step 1 (initialization)**

Problem is solved with the equivalent deterministic objective function and the constraint equation (21) is replaced with (17) for a fixed scalar *𝓏* to determine the set of new controlled variables. The algorithm starts by choosing a high value for the initial *z*-value as in (21), which makes the corresponding solution satisfy the demand with a probability level higher than*𝒫*_target_ = *β*_*m*_.

**Step 2 (MINLP solution)**

The problem is implemented with GAMS (General Algebraic Modeling System). The CPLEX is used to solve this deterministic problem.

**Step 3 (evaluate probability)**

Evaluate the multivariate normal probability (17). A subregion adaptive algorithm proposed by Genz and Bretz [24] is employed to carry out multivariate integration which makes this evaluation feasible.

**Step 4 (update*𝓏*-value)**

If the evaluated probability level is in the  *γ*  neighborhood of  *𝒫*_target_, the algorithm terminates since the goal of finding a schedule that satisfies the demand with a probability of *𝒫*_target_ is accomplished; otherwise the *z* value is updated and the previous steps are repeated to obtain another schedule.

To update the *𝓏*-value the following algorithm is used. The goal is to find a *𝓏*-value in (21) that provides a schedule such that the demand can be satisfied with a probability of *𝒫*_target_ = *β*_*m*_ over the entire time horizon. The *𝓏*-value needs to be obtained iteratively.

**Step 1 (obtain the upper and lower bounds of *𝓏*)**

First we choose *𝓏* = *𝓏*_*α*_ in (21). Obviously it yields a lower bound for the correct *𝓏*-value. We call it *𝓏*_lower_. We now run Step 2 of the algorithm for this lower bound and obtain an estimate of the probability with which the demand is being met. We call this probability *𝒫*_lower_. Next we choose an arbitrary large value for *𝓏*. We denote it by *𝓏*_upper_ and obtain a similar estimate of the probability of demand satisfaction. We denote this probability by *𝒫*_upper_.

**Step 2 (obtain the upper and lower bounds of *𝒫*_target_)**

In this step we obtain the upper percentiles of the univariate standard normal distribution for these probabilities and denote *𝒫*_upper_  and  *𝒫*_lower_ by *𝓏*_2_  and *𝓏*_1_, respectively. We also denote the corresponding percentile for the  *𝒫*_target_ value by  *𝓏*_target_.

**Step 3 (update *𝓏*-value)**

Based on these values the updated *𝓏*-value is obtained using the following linear interpolation formula:

(22) $$\begin{array}{c} {𝓏}_{\text{new}}={𝓏}_{\text{lower}}+\left(\frac{{𝓏}_{\text{target}}-{𝓏}_{1}}{{𝓏}_{2}-{𝓏}_{1}}\right)\left({𝒵}_{\text{upper}}-{𝒵}_{\text{lower}}\right). \end{array}$$

If the *𝒵*_new_ value is lower than *𝒵*_2_ and higher than *𝒵*_target_, then we replace  *𝒵*_2_  by*𝒵*_new_. If it is lower than*𝒵*_target_ and higher than *𝒵*_1_, we replace *𝒵*_1_ by *𝒵*_new_. We repeat this process using (17) until  *𝒫*_target_  is reached.

### 5.2. Update of the Planning and Scheduling Policy

As discussed in Section 3, the demand  *D* ^*M*^ ~ *N*(*μ*, Σ)  follows a multivariate normal distribution with a specific correlation structure. In order to decrease the uncertainty associated with the random variables *D*_*j*,*m*_^*M*^ and thus increase the predictive power of the stochastic model, it is proposed to update the corresponding schedule at the end of each macroperiod based on the realized production [17]. The update of the schedule is accomplished by recalculating the conditional multivariate probability function given the demand realizations in previous periods.

After solving the scheduling problem, only the decision variables associated with the immediately following period may be taken as final. The decision variables referring to successive periods can be used for planning and activities related to the operation.

The update of the planning policy and schedule at the end of macroperiod  *m*  is described in the following steps.

**Step 1 (update statistics)**

Update future demand statistics based on present and previous product demand realizations.

**Step 2 (resolve the problem)**

Resolve the scheduling problem for *m*′ = *m* + 1,…, *M*.

**Step 3 (update* m*)**

Set *m* ← *m* + 1. If *m* ≤ *M*, return to Step 1.

The first step allows the use of updated demand forecasting information. If there are no new demand forecasts available, the conditional multivariate probability density function based on the realizations of the demands in previous periods can be utilized. The procedure for finding the conditional distribution function is outlined below. Step 2 involves the solution of the problem, which, in turn, determines the new planning and scheduling policies.

The derivation of the conditional probability distribution based on the realization of previous random variables can be accomplished as follows. First, the random variable  *D*_*j*,*m*_^*M*^  is partitioned into two sets. The first set

(23) $$\begin{array}{c} P=\left\{D_{j,m^{\prime }}^{M}:1\leq m^{\prime }\leq m,m=1,\ldots ,M\right\} \end{array}$$

contains the crude mix demands realized in the past periods *m*′ = 1,…, *m*. The second set

(24) $$\begin{array}{c} F=\left\{D_{j,m^{\prime }}^{M}:m+1\leq m^{\prime }\leq M,i=1,\ldots ,M\right\} \end{array}$$

contains the uncertain crude mix demands for the future periods. Based on this partitioning, the variance-covariance matrix can be expressed as follows:

(25) $$\begin{array}{c} \Sigma =\left(\begin{array}{cc} \Sigma_{PP} & \Sigma_{PF}\\ \Sigma_{FP} & \Sigma_{FF} \end{array}\right), \end{array}$$

where  Σ_PP_  and  Σ_FF_  are the variance-covariance submatrices of the crude mix demands belonging to the sets *P* and* F*, respectively. The elements of submatrices Σ_PF_ = (Σ_FP_)^*T*^ are the covariances between the elements of *P* and *F*. Let

(26) $$\begin{array}{c} \Xi =\left(\xi_{i1},\xi_{i2},\ldots ,\xi_{it}\right) \end{array}$$

denote the realizations (outcomes) of the crude mix demands of set *P* associated with past periods. The conditional means of the uncertain future crude mix demands include

(27) $$\begin{array}{c} \mu_{F/P}=\mu_{F}+\Sigma_{FP}{\Sigma_{PP}}^{-1}\left(\Xi -\mu_{P}\right), \end{array}$$

where *μ*_*F*/*P*_ denotes the conditional demand expectations and  *μ*_*P*_  and  *μ*_*F*_ are the past and future mean values. The new (conditional) variance-covariance matrix is equal to

(28) $$\begin{array}{c} \Sigma_{F/A}=\Sigma_{FF}-\Sigma_{FP}{\Sigma_{PP}}^{-1}\Sigma_{FP}. \end{array}$$

A detailed treatise of conditional multivariate normal probability functions can be found in Tong, 1990 [18]. Note that while the conditional mean value  *μ*_*F*/*A*_ depends on the demand realizations in past periods, the conditional covariance matrix Σ_*F*/*A*_ is independent of the demand realizations  *Ξ*. Updating the probability distribution at the end of each period decreases the uncertainty (variances) associated with the remaining random variables, thus increasing the predictive power of the stochastic model. This is observed in the case study in Section 6.

## 6. Case Study

In the case study, three different crude mixes are to be produced. The detailed data of the case study is given in Table 1. The time horizon of 30 days is divided into 30 equal times. The schedule involves three macroperiods with 10 days. The product demands in each macroperiod are described by normal multivariate probability distributions. The expected (mean) values of the demands are given in Table 2, and their standard deviation is assumed to be 25% of their mean values.

The alternative model formulation is defined and solved using the proposed solution procedure.

Mode:

(29) CCOST=∑V=1NVCVUNLOAD(TvL−TvF)+∑v=1NVCVSEA(TvF−TvARR) +∑i=1NSTCiINVST∑t=1NSCH(Vi,tS+Vi,t−1S2) +∑j=1NBTCjINVBL∑t=1NSCH(Vj,tB+Vj,t−1B2) +∑t=1NSCH∑j=1NBT∑j′=1NBTCj,j′SETUP∑l=1NCDUZj,j′,l,t+∑j=1NBTCjPEN ×∑m=1NMmax⁡(0,Dj,mM−∑l=1NCDU∑TmTm+1−1Fj,l,tBC)Subject to:  Pr[∑l=1NCDU∑TmTm+1−1Fj,l,tBC≥DMj,m]≥βj,m[Pr⋂(j,m)∈Im(∑l=1NCDU∑TmTm+1−1Fj,l,tBC≥DMj,m)]≥βmothers  (1–7).

Model incorporates not only single but also joint chance constraints for setting probability targets for the satisfaction of a group of products. First the effect of correlation on the economic parameters and the optimal product mix is evaluated and discussed, and next the proposed update policy is applied on the example problem.

The problem is implemented with GAMS. The CONOPT and CPLEX 4.0 solvers are used to solve the formulated models above.

### 6.1. Use of Joint Probability Constraints

Two separate cases are considered for model involving (i) uncorrelated and (ii) arbitrarily correlated crude mix demands. For the correlated crude mix demands, a variance-covariance matrix is constructed and shown in (30). The matrix has sparsity of 60%, and its off-diagonal elements vary between −0.3 and 0.6. The bias toward positive covariance is introduced to maintain semipositive definiteness which is a property of all variance-covariance matrices. Consider

(30) $$\begin{array}{c} \Sigma =\left(\begin{array}{ccccccccc} 1.0 & 0.0 & -0.2 & 0.2 & 0.0 & 0.3 & 0.0 & 0.0 & 0.0\\ 0.0 & 1.0 & 0.0 & 0.0 & 0.3 & 0.0 & 0.0 & 0.6 & 0.2\\ -0.2 & 0.0 & 1.0 & 0.0 & 0.1 & 0.0 & 0.0 & 0.2 & 0.0\\ 0.2 & 0.0 & 0.0 & 1.0 & 0.0 & 0.0 & 0.0 & 0.2 & 0.0\\ 0.0 & 0.3 & 0.1 & 0.0 & 1.0 & 0.2 & 0.2 & 0.3 & 0.0\\ 0.3 & 0.0 & 0.0 & 0.0 & 0.2 & 1.0 & 0.0 & 0.2 & 0.1\\ 0.0 & 0.0 & 0.0 & 0.0 & 0.2 & 0.0 & 1.0 & 0.2 & 0.2\\ 0.0 & 0.6 & 0.2 & 0.2 & 0.3 & 0.2 & 0.2 & 1.0 & 0.0\\ 0.0 & 0.2 & 0.0 & 0.0 & 0.0 & 0.1 & 0.2 & 0.0 & 1.0 \end{array}\right). \end{array}$$

**Figure 2:**
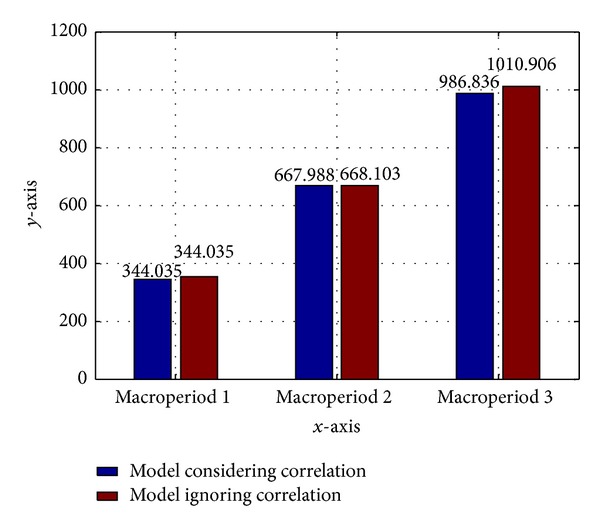
Comparison for the total operating cost with and without consideration of demand uncertainty.

The resulting MILP problem involves the objective function and a set of linear constraints representing the deterministic equivalent representation of the individual and joint probability constraints. The joint probability index  *β*_*m*_, for the set of all 9 crude oil demands, is set to 50%. For the value of  *β*_*m*_ is identified, the expectation of the cost is compared with those without considering demand correlation.  Figure 2 shows the minimization expected cost for the two cases. These results indicate that the expectation of the total cost in the model considering the correlation decreases.

The lower expectation of the total cost results from the fact that the joint probability target can be met with smaller production levels than the ones ignoring correlations.

### 6.2. Revision of Planning Policy

Using the correlation matrix in (30), a demand realization is randomly generated using the method presented by Tong (1980) [18]. The crude mix demands realizations are given in Table 3; a joint probability target of 50% is set to be the demand satisfaction of each crude mix in all time periods. This introduces joint probability constraints in the following form:

(31) $$\begin{array}{c} \text{Pr}\left[\underset{\left(j,m\right)\in I_{m}}{⋂}\left(\sum_{l=1}^{N^{\text{CDU}}}\sum_{T_{m}}^{T_{m+1}-1}F_{j,l,t}^{\text{BC}}\geq D_{j,m}^{M}\right)\right]\geq 0.5. \end{array}$$

The resulting optimization problem is solved in the entire time horizon. Next, based on (i) the demand realizations in the first period, (ii) the correlation matrix, (iii) the mean values of the remaining random demands, and (iv) the remaining inventory at the beginning of the second period, a new planning revision problem is solved for macroperiod  *m* ∈ (2-3). This is repeated for all remaining macroperiods. The updated values of the mean vectors, standard deviations, and covariance matrix after each time period are given in Tables 4, 5, and 6.

**Figure 3:**
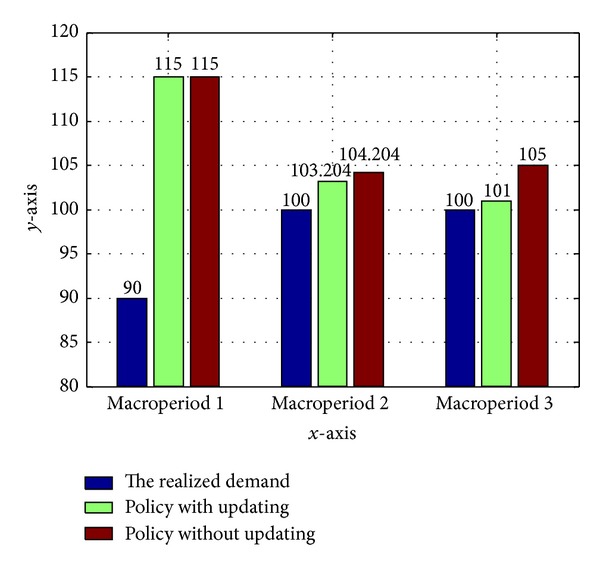
Comparison for the realized demand and the actual production for crude mix under three circumstances.

Two separate cases are considered for the models involving (i) correlated crude mix demands using the proposed policy and (ii) correlated crude mix demands without the policy. The actual production amount for crude mix 1 is shown in Figure 3. Examination of the data reveals that the actual production amount using the updating policy is close to the actual realized demand.

Clearly, when the solution is updated, the planning policy is much more effective because overproduction is decreased. The proposed scheduling updating policy by updating the probability distribution at the end of each period decreased uncertainty associated with the remaining random variables and thus increased the predictive power of the stochastic model for the subsequent periods.

## 7. Conclusion

In this paper, the multiperiod crude oil scheduling problem under demand uncertainty was addressed. A stochastic two-level time structure model considering the uncertain crude mix demand correlations is formulated. The model involves the minimization of the expected cost subject to constraints for the satisfaction of single- and/or multiple-crude mix demands with a prespecified level of probability. A chance constrained programming formulation is developed for solving the problem, and the deterministic form of the stochastic constraint is used to solve the problem in an iterative way. Moreover, a revision strategy for the update of the corresponding schedule at the end of each time period based on the realized production was also presented. This was accomplished by recalculating the conditional multivariate probability function given the demand realizations in previous periods.

Results on the correlated case study demonstrate that the expected profit and in particular the corresponding planning policy and schedule are strongly affected by the presence of correlations. The proposed planning revision and scheduling updating policy by updating the probability distribution at the end of each period decreased uncertainty associated with the remaining random variables and thus increased the predictive power of the stochastic model for the subsequent periods.

## Figures and Tables

**Table 1:** System information.

Scheduling horizon			30		
Number of vessel arrivals			9		
Vessel	Arrival time	Amount of crude	Concentration of key component 1		Concentration of key component 2

Vessel 1	1	100	0.01		0.04
Vessel 2	4	100	0.03		0.02
Vessel 3	7	100	0.05		0.01
Vessel 4	11	100	0.01		0.04
Vessel 5	14	100	0.03		0.02
Vessel 6	17	100	0.05		0.01
Vessel 7	21	100	0.01		0.04
Vessel 8	24	100	0.03		0.02
Vessel 9	27	100	0.05		0.01

Number of storage tanks			3		

Storage tank		Capacity	Initial oil amount	Initial concentration of key component 1	Initial concentration of key component 2

Storage tank 1		100	20	0.0167	0.0333
Storage tank 2		100	50	0.03	0.0225
Storage tank 3		100	70	0.0433	0.0133

Number of charging tanks			3		

Charging tank		Capacity	Initial oil amount	Initial concentration of key component 1	Initial concentration of key component 2

Charging tank 1		100	30	0.0167	0.0333
Charging tank 2		100	50	0.03	0.0225
Charging tank 3		100	30	0.0433	0.0133

Number of CDUs				2	
Costs involved in vessel operation				Unloading cost: 8, sea waiting cost: 5	
Tank inventory costs				Storage tank: 0.05; charging tank: 0.08	
Changeover cost for charged oil switch				50	
The penalty cost for crude mix shortfalls				0.5	

**Table 2:** Mean values of the product demand.

Macroperiod	Product 1	Product 2	Product 3
1	90	98	95
2	100	80	98
3	100	90	98

**Table 3:** Employed realizations of the random product demands.

Macroperiod	Product 1	Product 2	Product 3
1	92.5504	107.0773	83.6901
2	104.2967	82.7651	92.1586
3	98.5225	93.5261	114.0730

**Table 4:** Updates of the product demand expectations during the process of policy revision.

Policy revision	Macroperiod *M *	Product 1	Product 2	Product 3
1	1	90	98	95
	2	100	80	98
	3	100	90	98
2	2	100.0438	81.0850	98.0650
	3	100	92.3	99.6155
3	3	100.7265	92.1073	98.6984

**Table 5:** Updates of the product demand standard deviations during the process of policy revision.

Policy revision	Macroperiod *M *	Product 1	Product 2	Product 3
1	1	4.7434	4.9497	4.8734
	2	5	4.4721	4.9497
	3	5	4.7434	4.9497
2	2	4.8947	4.2417	4.7120
	3	5.0	3.6691	4.8497
3	3	4.8821	3.3937	4.8020

**Table 6:** Updates of the product demand correlation matrix.

Policy revision									
1	1.0	0.0	−0.2	0.2	0.0	0.3	0.0	0.0	0.0
	0.0	1.0	0.0	0.0	0.3	0.0	0.0	0.6	0.2
	−0.2	0.0	1.0	0.0	0.1	0.0	0.0	0.2	0.0
	0.2	0.0	0.0	1.0	0.0	0.0	0.0	0.2	0.0
	0.0	0.3	0.1	0.0	1.0	0.2	0.2	0.3	0.0
	0.3	0.0	0.0	0.0	0.2	1.0	0.0	0.2	0.1
	0.0	0.0	0.0	0.0	0.2	0.0	1.0	0.2	0.2
	0.0	0.6	0.2	0.2	0.3	0.2	0.2	1.0	0.0
	0.0	0.2	0.0	0.0	0.0	0.1	0.2	0.0	1.0
2				1	−0.0045	−0.0671	0	0.2531	0
				−0.0045	1	0.2146	0.2109	0.1352	−0.0646
				−0.0671	0.2146	1	0	0.2546	0.1072
				0	0.2109	0	1	0.2586	0.2041
				0.2531	0.1352	0.2546	0.2586	1	−0.1583
				0	−0.0646	0.1072	0.2041	−0.1583	1
3							1.0000	0.2672	0.2312
							0.2672	1.0000	−0.1970
							0.2312	−0.1970	1.0000

## References

[1] Pinto JM, Joly M, Moro LFL (2000). Planning and scheduling models for refinery operations. *Computers and Chemical Engineering*.

[2] Li J, Karimi IA, Srinivasan R (2010). Recipe determination and scheduling of gasoline blending operations. *AIChE Journal*.

[3] Li J, Misener R, Floudas CA (2012). Continuous-time modeling and global optimization approach for scheduling of crude oil operations. *AIChE Journal*.

[4] Jia Z, Ierapetritou M, Kelly JD (2003). Refinery short-term scheduling using continuous time formulation: Crude-oil operations. *Industrial and Engineering Chemistry Research*.

[5] Lee H, Pinto JM, Grossmann IE, Park S (1996). Mixed-integer linear programming model for refinery short-term scheduling of crude oil unloading with inventory management. *Industrial and Engineering Chemistry Research*.

[6] Wenkai L, Hui C-W, Hua B, Tong Z (2002). Scheduling crude oil unloading, storage, and processing. *Industrial and Engineering Chemistry Research*.

[7] Reddy PCP, Karimi IA, Srinivasan R (2004). Novel solution approach for optimizing crude oil operations. *AIChE Journal*.

[8] Chryssolouris G, Papakostas N, Mourtzis D (2005). Refinery short-term scheduling with tank farm, inventory and distillation management: an integrated simulation-based approach. *European Journal of Operational Research*.

[9] Hu Y, Zhu Y (2007). An asynchronous time slot-based continuous time for mulation approach for crude oil scheduling. *Computers and Applied Chemistry*.

[10] Jia Z, Ierapetritou M (2004). Efficient short-term scheduling of refinery operations based on a continuous time formulation. *Computers and Chemical Engineering*.

[11] Jia Z, Ierapetritou M, Kelly JD (2003). Refinery short-term scheduling using continuous time formulation: crude-oil operations. *Industrial and Engineering Chemistry Research*.

[12] Neiro SM, Pinto JM Supply chain optimization of petroleum refinery complexes.

[13] Wang J, Rong G (2010). Robust optimization model for crude oil scheduling under uncertainty. *Industrial and Engineering Chemistry Research*.

[14] Cao C, Gu X (2006). Chance constrained programming with fuzzy parameters for re_nery crude oil scheduling problem. *Fuzzy Systems and Knowledge Discovery*.

[15] Cao C, Gu X, Xin Z (2009). Chance constrained programming models for refinery short-term crude oil scheduling problem. *Applied Mathematical Modelling*.

[16] Cao C, Gu X, Xin Z (2010). Stochastic chance constrained mixed-integer nonlinear programming models and the solution approaches for refinery short-term crude oil scheduling problem. *Applied Mathematical Modelling*.

[17] Petkov SB, Maranas CD (1997). Multiperiod Planning and Scheduling of Multiproduct Batch Plants under Demand Uncertainty. *Industrial and Engineering Chemistry Research*.

[18] Tong YL (1980). *Probability Inequalities for Multivariate Distributions*.

[19] Fleischmann B, Meyr H (1997). The general lotsizing and scheduling problem. *OR Spectrum*.

[20] Charnes A, Cooper WW (1962). Normal deviates and chance constraints. *Journal of the American Statistical Association*.

[21] Ozturk UA, Mazumdar M, Norman BA (2004). A solution to the stochastic unit commitment problem using chance constrained programming. *IEEE Transactions on Power Systems*.

[22] Billingsley P (1986). *Probability and Measure*.

[23] Lee FN, Lin M-Y, Breipohl AM (1990). Evaluation of the variance of production cost using a stochastic outage capacity state model. *IEEE Transactions on Power Systems*.

[24] Genz A, Bretz F (2002). Comparison of methods for the computation of multivariate t probabilities. *Journal of Computational and Graphical Statistics*.

